# Treatment of chronic kidney diseases with histone deacetylase inhibitors

**DOI:** 10.3389/fphys.2015.00121

**Published:** 2015-04-28

**Authors:** Na Liu, Shougang Zhuang

**Affiliations:** ^1^Department of Nephrology, Shanghai East Hospital, Tongji University School of MedicineShanghai, China; ^2^Department of Medicine, Rhode Island Hospital and Alpert Medical School, Brown UniversityProvidence, RI, USA

**Keywords:** histone deacetylases, chronic kidney diseases, renal fibrosis, renal fibroblasts

## Abstract

Histone deacetylases (HDACs) induce deacetylation of both histone and non-histone proteins and play a critical role in the modulation of physiological and pathological gene expression. Pharmacological inhibition of HDAC has been reported to attenuate progression of renal fibrogenesis in obstructed kidney and reduce cyst formation in polycystic kidney disease. HDAC inhibitors (HDACis) are also able to ameliorate renal lesions in diabetes nephropathy, lupus nephritis, aristolochic acid nephropathy, and transplant nephropathy. The beneficial effects of HDACis are associated with their anti-fibrosis, anti-inflammation, and immunosuppressant effects. In this review, we summarize recent advances on the treatment of various chronic kidney diseases with HDACis in pre-clinical models.

## Introduction

Histone and non-histone protein acetylation has been widely studied in the field of cancer research (Kwon et al., 2009; Mahalingam et al., 2010; Selinger et al., 2011). Histone acetyltransferases (HATs) and histone deacetylases (HDACs) can mediate the acetylated/deacetylated states of histones (Bush and McKinsey, 2010). HATs induce acetylation of histones H3 and H4 on lysine amino groups (Spencer and Davie, 1999) whereas HDACs remove acetyl groups from the acetylated proteins. Acetylation of the ε-amino groups of lysine residues in nucleosomal histone tails by HATs is considered necessary for the chromatin structure to relax, allowing activation of transcriptional activators and initiation of gene induction. Inhibition of HDACs with HDAC inhibitors (HDACis) also enhances the deposition of acetylated histones H3 and H4, thereby modifying chromatin structure and regulating gene transcription (Turner, 1993; Van Lint et al., 1996). In addition, HDACs are able to catalyze deacetylation of many non-histone proteins, thus, they are also called lysine deacetylases to describe their functions more precisely (Glozak et al., 2005). HDACis have been reported to regulate gene transcription positively or negatively in a gene-specific manner (Marks et al., 2000).

HDACs are divided into four groups, mainly according to the homology of yeast HDACs. Class I HDACs (HDAC1, 2, 3, and 8) are critically connected with yeast RPD3 gene. Class II HDACs (HDAC4, 5, 6, 7, 9, and 10) are related to yeast Hda1 gene. Class III HDACs (SIRT1-7) are homologous to silent information regulator 2 (Sir2) and have no sequence similarity to class I and II HDACs; these Sir2 proteins, also called sirtuins are unaffected by known class I/II HDACis. Class IV (HDAC11) has conserved residues in its catalytic regions that are shared by both class I and II HDACs. Class I and II HDACs need Zn^2+^ for their enzymatic reaction. Class IV also has a Zn^2+^ based reaction mechanism. However, class III HDACs do not require Zn^2+^ for their catalysis, but strictly depend on the cofactor NAD^+^ (Pang and Zhuang, 2010).

Currently, the expression profiles and distribution of HDACs in the kidney have not been completely clarified. It has been documented that class I HDAC isoforms are expressed in the cortex of developmental kidney, renal fibroblasts, and renal tubular cells (Pang et al., 2011; Chen and El-Dahr, 2013; Tang et al., 2013). HDAC5 and 6 have been identified in the renal tubules (Marumo et al., 2008; Liu et al., 2012b). SIRT1 is expressed in both renal tubules (Zhou et al., 2013) and fibroblasts (Ponnusamy et al., 2014) and HDAC11 is expressed in renal tubules (Kim et al., 2013). HDACs have been shown to be involved in a variety of cellular functions such as proliferation, survival, differentiation, and immunological responses (Van Beneden et al., 2013).

Most functional roles of HDACs are revealed by application of HDACis, which are classified into four categories according to chemical structures: hydroxamates (e.g., vorinostat), cyclic peptides (e.g., romidepsin), aliphatic acids (e.g., phenylbutyrate), and benzamides (entinostat) (Miller et al., 2003; Marks et al., 2004; Dokmanovic and Marks, 2005; Ma et al., 2009). HDACis primarily target the zinc domains to exert the biological effects through cell cycle arrest, differentiation and apoptosis in a variety of tumor models (Acharya et al., 2005). Treatment of renal cell carcinoma with HDACis also resulted in tumor growth inhibition (Ramakrishnan and Pili, 2013). To date, two HDACis, vorinostat, and romideps, have been approved by the FDA to treat cutaneous and peripheral T cell lymphoma. Other 20 different HDACis have been tested in the clinic (West and Johnstone, 2014). The common side-effects of HDACis in humans include fatigue, nausea, and vomiting and resolve upon treatment withdrawal (Minucci and Pelicci, 2006).

**Figure 1:**
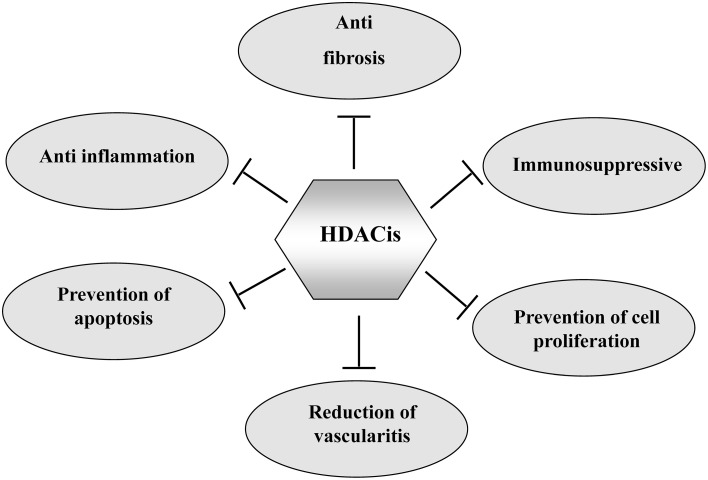
**The mechanisms by which HDACIs attenuate chronic kidney diseases**. HDACIs can protect against chronic kidney diseases through multiple mechanisms as indicated.

Although the tumor has been the primary target for HDACis, HDAC inhibition has also shown beneficial effects in some non-neoplastic disorders. HDACis are effective in attenuating the pathogenesis of several forms of chronic kidney disease and improving renal function. In this article, we highlight the therapeutic application of HDACis in pre-clinical models of renal injury and discuss the mechanisms involved (Figure 1).

## HDACs in renal interstitial fibrosis

Progression and development of renal injuries ultimately leads to renal interstitial fibrosis (Neilson, 2006; Wynn, 2008). Kidney fibrosis is characterized with activation and proliferation of renal interstitial fibroblasts as well as accumulation of extracellular matrix (ECM) components. During development of renal fibrosis, multiple cytokine, and growth factor signaling pathways are activated and involved in this process. Emerging evidence indicates that HDACs are also implicated in renal fibrogenesis. The mechanisms by which HDACs mediates renal fibrogenesis remain elusive, but may be associated with regulating the expression of inflammatory and profibrotic genes and activation of cell signaling pathways that mediate renal fibrosis. In the earlier studies, Pang et al. (2009) demonstrated that treatment with trichostatin A (TSA), a pan HDACi that can block both class I and class II HDACs, attenuates renal fibrosis in a murine model of unilateral ureteral obstruction (UUO). TSA treatment also significantly inhibits expression of α-SMA and fibronectin, two hallmarks of activated fibroblasts (Pang et al., 2009). Moreover, silencing of HDAC1 or HDAC2 using specific siRNA blocked renal fibroblast proliferation and reduced phosphorylation of STAT3 (signal transducer and activator of transcription 3), a signaling molecule associated with proliferation of renal fibroblasts and development of renal fibrosis (Pang et al., 2011). Recently, Manson et al. (2014) demonstrated that TSA treatment preserves the expression of Bmp-7 transcription and attenuates the pathogenesis of renal injury in obstructive nephropathy. As BMP-7 is a potent anti-fibrotic molecule, restoration of BMP-7 expression by TSA represents another mechanism by which HDACis protect against chronic kidney injury. In addition, Marumo et al. (Liu et al., 2013) showed that HDAC inhibition also alleviates renal fibrosis through suppression of inflammatory responses in the injured kidney.

Since non-selective blockade of class I and class II HDACs does not allow elucidation of their individual roles in renal fibrogenesis, Liu et al. (2013) further examined the effect of MS-275, a selective class I inhibitor, on UUO injury and renal interstitial fibroblast activation and proliferation. Administration of MS-275 inhibited both renal fibroblast activation and proliferation and attenuated progression of kidney fibrosis. MS-275 treatment also inhibited UUO-induced production of TGF-β 1 and phosphorylation of Smad3 and EGFR (epidermal growth factor receptor). These results suggest that specific blockade of only class I HDAC can inhibit renal fibrogenesis through a mechanism involved in the inactivation of the TGF-β 1/Smad3 and EGFR signaling pathways. Although there is no study thus far to compare the pharmacological effect of TSA and MS-275 on renal fibroblast activation and development of renal fibrosis, it has been reported that pre-treatment with either valproic acid, another inhibitor of class I or TSA attenuates glomerulosclerosis and tubulointerstitial fibrosis to the similar degree. Delayed administration of these two inhibitors also showed comparable effects on the inhibition of renal fibrosis (Van Beneden et al., 2013). Thus, it appears that class I HDACs play a pre-dominant role in regulating renal fibrogenesis. Class III HDACs have been implicated in the regulation of renal fibrosis. It is evident that specific inhibition of SIRT1/2 alleviates progression of renal fibrogenesis and reduces renal fibroblast activation (Ponnusamy et al., 2014). Mechanistic studies showed that blocking SIRT1/2 inhibits activation of epidermal growth factor receptor (EGFR) and platelet derived growth factor receptors (PDGFR), two growth factor receptors associated with renal fibrosis (Chen et al., 2011; Liu et al., 2012a).

Collectively, these studies indicate that HDACs contribute to renal fibroblast activation and fibrogenesis. Additional studies are needed to clarify the role of individual HDAC isoforms in mediating these processes and elucidate the mechanisms involved in a great detail.

## HDACs in polycystic kidney diseases

Autosomal dominant polycystic kidney disease (ADPKD) is very common hereditary kidney diseases in humans, affecting 1/500 in the United States (Gabow, 1993). In ADPKD patients, a large number of bilateral kidney cysts displace normal kidney parenchyma, leading to end-stage renal disease (ESRD). ADPKD is mainly caused by gene mutations in one of two genes: PKD1, which accounts for approximately 85–95% of the cases and PKD2, which affects about 5–15% of the cases (Peters and Sandkuijl, 1992). ADPKD is characterized by development of multiple bilateral renal cysts, and increased renal epithelial cell proliferation and fluid secretion. As the gene product of *PKD2*, polycystin-2 (PC2), either alone or in complex with the gene product of *PKD1*, polycystin-1 (PC1), functions as a calcium-permeable cation channel and regulates intracellular Ca^2+^ levels, alteration of signaling pathways regulated by calcium such as cAMP-dependent B-Raf and ERK (extracellular signal-regulated kinase) activation resulted in abnormal proliferation of tubule epithelial cells (Yamaguchi et al., 2006). In addition, activation of many other signaling pathways and transcription factors such as EGFR and p53 is also involved in the development and growth of polycystic kidneys (Harris and Torres, 2014).

Emerging evidence has revealed the regulatory role of HDACs in the pathogenesis of polycystic kidneys. Xie et al., showed (Xia et al., 2010) that histone deacetylase 5 (HDAC5) is a target of polycystin-dependent fluid stress sensing in renal epithelial cells in mice. Stimulation of polarized epithelial monolayers with fluid flow induced phosphorylation and nuclear export of HDAC5 whereas dwonregulation of HDAC5 or treatment with TSA reduced cyst formation in *Pkd2*^−/−^ mouse embryos. Cao et al. (2009) demonstrated that TSA treatment can affect both body curvature and laterality, two pathological changes associated with cyst formation in zebrafish and block cyst formation in pkd2 knockdown animals. Treatment with valproic acid (VPA), a class I specific HDACi, also delays the development of cyst production and improves renal function in a mouse ADPKD model. In addition, Fan et al. showed that administration of TSA in pregnant mice prevented cyst formation in *Pkd1* mutant embryonic kidneys (Fan et al., 2012). TSA treatment can ameliorate p53-induced repression of the PKD1 expression, Chang et al. (2006) and Thivierge et al. (2006). As EGFR activation and nuclear translocation of β-catenin are essential for ADPKD, the role of HDAC6 in regulating these biological responses was examined. HDAC6 inhibition blocks EGF-induced β-catenin nuclear localization, leading to inhibition of epithelial cell proliferation and promotion of EGFR degradation (Li et al., 2008). These studies suggest that class I/II HDAC activation is essential for PKD development and that HDACis may be possible drug treatments for PKD.

A recent study further reveals that SIRT is also involved in the pathogenesis of ADPKD (Zhou et al., 2013). SIRT1 upregulation was observed in embryonic and post-natal Pkd1-mutant mouse renal epithelial cells and tissues whereas double conditional knockouts of PKD1 and SIRT1 as well as inhibition of SIRT1 with a pan-sirtuin inhibitor (nicotinamide) or a SIRT1-specific inhibitor (EX-527) resulted in delayed renal cyst formation. Silence or inhibition of SIRT1 also reduced renal epithelial cell proliferation, but potentiated apoptosis. Further studies show that SIRT1 mediates cystic epithelial cell proliferation through altering retinoblastoma (RB) protein acetylation/phosphorylation and promotes their survival via p53 deacetylation. This study elucidates a functional role of SIRT1 in regulating ADPKD and provides a molecular basis for using SIRT1 inhibitors to interfere with cyst formation (Zhou et al., 2013).

## HDACs in diabetic nephropathy

Diabetic nephropathy (DN) is characterized by ECM protein accumulation in glomerular mesangium and tubulointerstitium with thickening of glomerular and tubular basement membranes, ultimately progressing to glomerulosclerosis and tubulo-interstitial fibrosis (Mauer et al., 1984). The earliest finding of renal involvement in DN is glomerular hypertrophy, which is caused by glomerular hyper-filtration. Although targeting diverse signaling pathways has been reported to attenuate the pathogenesis of DN, two animal studies have demonstrated the inhibitory effect of HDACis on DN. Gilbert et al. showed that vorinostat administration resulted in attenuation of renal hypertrophy in rats (Gilbert et al., 2011). Advani et al. demonstrated that vorinostat was effective in decreasing albuminuria and mesangial matrix accumulation in streptozotocin–wild-type mice (Advani et al., 2011). *In vitro*, treatment with VPA and SK-7041, two class I-selective HDACis, can also reduce expression of ECM components in renal epithelial cells (NRK52-E) (Noh et al., 2009). In addition, HDAC2 activity was upregulated in the kidneys of strotozotocin (STZ) induced diabetic rats and db/db mice. Treatment with N-acetylcysteine, an antioxidant, decreased TGF-β 1 mediated activation of HDAC2 in NRK52-E cells (Noh et al., 2009). These data suggest that HDACs are required for the development of DN and that reactive oxygen species may play an essential role in mediating TGF-β 1-induced activation of HDAC2.

EGFR activation has been shown to be implicated in the DN (Chen et al., 2014; Zhang et al., 2014). To understand whether EGFR expression is associated with the HDAC activity, Gilbert et al. (2011) further investigated the effect of vorinostat on the expression of EGFR in the early stage of diabetes. They found that daily treatment with vorinostat in diabetic rats for 4 weeks remarkably reduced EGFR expression and subsequently inhibited kidney growth and glomerular hypertrophy. In cultured rat proximal tubule cells, treatment with vorinostat also decreased EGFR expression, concomitant with cellular proliferation inhibition. Therefore, HDACs may regulate early DN through activation of the EGFR signaling pathway.

Podocyte damage accelerates the development of DN, characterized by loss of cytoskeleton protein integrity, such as nephrin. An early study showed that miR-29a is a potent regulator that inhibits fibrotic matrix expression in high glucose–stressed renal proximal tubule cells (Du et al., 2010). A recent study (Lin et al., 2014) indicated that miR-29a is protective against diabetes-induced podocyte damage, glomerular fibrosis and inflammation, and renal dysfunction. However, HDAC4-dependent H3K9 hypoacetylation counteracts miR-29a transcription in high glucose–stressed podocytes, suggesting that HDAC4 may be an important mediator in diabetic podocytopathy. Indeed, stimulation of podocytes with high glucose, advanced glycation end products, or transforming growth factor-β can increase HDAC4 expression and specific silencing of HDAC4 reduces podocyte injury in streptozotocin-induced diabetic rats and diabetic db/db mice (Wang et al., 2014). Further studies showed that the protective effect of HDAC4 inhibition is associated with prevention of autophagy defects and suppression of renal inflammation (Wang et al., 2014). Therefore, HDAC4 is a critical epigenetic mediator in the pathogenesis of DN, and specific inhibition of HDAC4 could serve as a therapeutic approach for DN and related renal diseases.

While application of HDACis is effective in the attenuation of DN, the combination of HDACis with other inhibitors might have additive or synergistic effects. Although such studies have not been performed in the DN, the combination of an ACE inhibitor with a HDACi has been reported to provide a better renal protection in a mouse model of HIV-associated nephropathy (Zhong et al., 2013). These two inhibitors can affect several important pathways involved in kidney inflammation and fibrosis, such as NF-κ B, interleukin-1, TGF-β, mitogen-activated protein kinase, and apoptosis signaling (Zhong et al., 2013). Thus, examination of the therapeutic effect of HDACis in the treatment of DN in combination with other drugs is warranted.

## HDACs in lupus nephritis

Systemic lupus erythematosus (SLE) is a very common autoimmune disease (Alderaan et al., 2015). Two studies have examined the role of HDACs in the pathogenesis of SLE in the MRL-lpr/lpr murine model of lupus. Mishra et al. (2003) demonstrated a remarkable reduction in proteinuria, glomerulonephritis, and spleen weight after treatment with TSA. TSA was also effective in the downregulation of IL-12, IFN-γ, IL-6, and IL-10 expression levels in splenocytes of this model. Regna et al. showed that HDAC inhibition with ITF2357, a specific inhibitor of class I and II HDAC, reduces sera and urinary markers of lupus, and suppresses expression of several inflammatory cytokines (IL-1β, TNF-α, IL-6, and IFN-γ) and improves kidney histopathology (Regna et al., 2014). These data suggest that class I and II HDACs contribute to the development of lupus and application of HDACis may have therapeutic benefits in the treatment of SLE.

## Aristolochic acid nephropathy

Aristolochic acid nephropathy is a progressive renal interstitial fibrosis, frequently associated with urothelial malignancies (Debelle et al., 2008). Recently, Novitskaya et al. (2014) examined the effect of a HDACi, 4-(phenylthio)butanoic acid (PTBA) analog methyl-4-(phenylthio)butanoate (M4PTB), on the kidney injury induced by aristolochic acid. They found that treatment with M4PTB promotes renal recovery and reduces renal fibrosis after aristolochic acid injury in mice. These beneficial effects are associated with increased renal tubular cell proliferation and decreased G2/M arrest of regenerating renal tubular epithelial cells. Furthermore, M4PTB treatment decreased peritubular macrophage infiltration and expression of macrophage chemokines such as CX3Cl1 and CCL2. Since an increased number of renal epithelial cell arrested at G2/M phase of cell cycle represents a maladaptive repair process that leads to renal fibrosis (Yang et al., 2010), class I/II HDACs may contribute to the development of CKD after aristolochic acid injury.

## HDACs in transplant kidney injury

Calcineurin inhibitors (CNIs) decrease the rate of acute rejection in renal transplantation patients, but side effects such as nephrotoxicity, neurotoxicity, and diabetogenicity (Shapiro et al., 1994), limit application of these drugs. Furthermore, CNIs are less effective in preventing chronic allograft rejection and the promotion of tolerance. Therefore, it is essential to search for novel and safer immunosuppressants with different mechanisms. Histone deacetylases (HDACs) are known to mediate transcription of genes that trigger immunological responses (Johnstone, 2002; Remiszewski, 2002). Studies examining the effect of HDACis on kidney injury after transplantation suggest that HDACis can initiate immunosuppression and prolong graft survival (Takahashi et al., 1996; Mishra et al., 2001). Edens et al. showed that HDACis were able to induce antigen-specific energy in lymphocytes (Edens et al., 2006). Tao et al. reported that HDAC inhibition improved the generation and function of regulatory T cells (Tao and Hancock, 2007). Moreover, Kinugasa et al. demonstrated that FR276457 had a remarkable immunosuppressive effect in a heterotopic cardiac transplant rat model (Kinugasa et al., 2008) and was able to prolong the median survival time (MST) in transplanted grafts in a canine renal transplant model. The combination of FR276457 with tacrolimus can also prevent allograft rejection. Histopathological analysis indicated that FR276457 suppressed mononuclear cell infiltration and vasculitis. Therefore, HDAC inhibition may prolong the MST in transplanted grafts when administered alone or combined with other immunosuppressive agents.

## Conclusion and future directions

Numerous studies have shown that treatment with HDACis is able to inhibit activation and proliferation of cultured renal interstitial fibroblasts and attenuate renal fibrosis in animal models. HDAC inhibition is also beneficial for other chronic kidney diseases caused by the diverse etiologies, as listed in Table 1. Given the large number of distinct HDACs, most studies in this field are currently conducted by using pan-HDACis or class-specific HDACis. Thus, there is a need to identify specific functions of each HDAC and to develop small molecule inhibitors that can selectively modulate the activities of individual HDAC isoforms. In addition, it is necessary to clarify the profile of HDAC-modulated proteins in the setting of renal fibrosis and other kidney diseases by using some novel techniques such as proteomics to globally analyze protein lysine acetylation in response to HDAC inhibition. Although numerous clinical trials for application of HDACIs in tumors have been reported, there is no clinical trial thus far to test the therapeutic effects of those inhibitors in patients with CKD. Therefore, further investigation of the mechanisms, efficacy and toxicity of HDACis in the pre-clinical model of CKD will be helpful for initiating clinical trials to assess the feasibility of HDACis in the treatment of this disease in the future.

**Table 1:** **Effects of HDAC inhibitors on experimental kidney disorders**.

**Disease models**	**HDAC inhibitors**	**Selectivity**	**Effects of HDAC inhibitors**	**Mechanisms**	**References**
Renal interstitial fibrosis	TSA	HDAC I/II	Attenuates renal fibroblast proliferation, α-SMA, fibronectin expression	Inhibits STAT3 activation induced by UUO	Pang et al., 2009, 2011
	Sodium valproate	HDACI	Attenuates macrophage infiltration and fibrotic changes	Reduces CSF-1 expression induced by TNF-α in renal tubular cells Inhibits TGF-β /Smad3 and EGFR signaling	Marumo et al., 2010
	MS-275	HDACI	Inhibits renal fibroblast activation	Inhibits TGF-β /Smad3 and EGFR signaling	Liu et al., 2013
	TSA, VPA	HDAC I/II	Hamperes glomerulosclerosis and tubulointerstitial fibrosis	N/A	Van Beneden et al., 2013
	Sirtinol	SIRT1/2	Inhibits renal fibroblast activation and proliferation as well as renal fibrogenesis	Inhibits EGFR and PDGFRβ signaling.	Ponnusamy et al., 2014
Polycystic kidney diseases	TSA	HDAC I/II	Attenuates p53 induced repression of the PKD1 promoter	Deacetylates p53 and binds with Sp1	Van Bodegom et al., 2006
	TSA	HDAC I/II	Reduces cyst formation	N/A	Xia et al., 2010
	TSA, VPA	HDACI/II	Suppress kidney cyst formation	N/A	Cao et al., 2009
	EX-527	SIRT1	Delays renal cyst formation	Inhibits cystic epithelial cell proliferation and induces cystic epithelial cell apoptosis	Zhou et al., 2013
Diabetic nephropathy	TSA, VPA, SK7041	HDAC I/II HDAC I	Attenuate ECM accumulation and EMT	Suppresses TGF-β 1 induced HDAC2 activation	Noh et al., 2009
	Vorinostat	HDAC I/II	Attenuates cellular proliferation, blunts renal growth, and glomerular hypertrophy	Downregulates EGFR expression	Gilbert et al., 2011
	SAHA	HDAC I/II	Decreases albuminuria, mesangial collagen IV deposition, and oxidative-nitrosative stress	Reduces eNOS expression in mouse kidneys and in cultured human umbilical vein endothelial cells	Advani et al., 2011
	Sodium butyrate	Pan HDAC inhibitor	Improves renal function	Inhibits apoptosis and DNA damage	Khan and Jena, 2014
Lupus nephritis	TSA, SAHA	HDAC I/II	Reduces proteinuria, glomerulonephritis and spleen weight	Downregulates IL-12, IFN-γ, IL-6, and IL-10 expression	Mishra et al., 2003
	ITF2357	HDAC I/II	Improves kidney histopathology	Suppresses expression of IL-1β, TNF-α, IL-6, and IFN-γ	Regna et al., 2014
Aristolochic acid nephropathy	PTBAs	Pan HDAC inhibitor	Accelerate recovery and reduce post-injury fibrosis	Decrease G2/M arrest and reduce macrophage infiltration	Novitskaya et al., 2014
Transplant kidney injury	FR276457	Pan HDAC inhibitor	Prolongs allograft survival	Suppresses mononuclear cell infiltration and vasculitis, and inhibits the proliferation of Jurkat cells by targeting activity of NF-κ B.	Kinugasa et al., 2009

*CSF-1, colony stimulating factor 1; EGFR, epidermal growth factor receptor; HDAC, histone deacetylase; PTBA, 4-(phenylthio)butanoic acid; SAHA, suberoylanilide hydroxamic acid; STAT3, signal transducer and activator of transcription 3; α-SMA, α-smooth muscle actin; TSA, Trichostatin A; VPA, valproic acid; TNF-α, tumor necrosis factor; TGF-β, transforming growth factor-β*.

### Conflict of interest statement

The authors declare that the research was conducted in the absence of any commercial or financial relationships that could be construed as a potential conflict of interest.
